# Dentists in the Army and Navy

**Published:** 1911-10-15

**Authors:** Joseph D. Eby

**Affiliations:** Atlanta, Ga.


### DENTISTS IN THE ARMY AND NAVY.

BY JOSEPH D. EBY, D.D.S., ATLANTA, GA.

Read before the Tennessee State Dental Association, May 25, 1911.

Following the second consecutive defeat of the "Bill" Act to create an Army Dental Corps with rank, which "Act" has been repulsed practically and largely by the opposition and influence of the Medical Department, at Washington, there appears the appended article, (first published from the Tribune Bureau, Washington, D. C., February 16th, 1911), the authorship of which is only too obvious from beginning to end.

The article appears under the head lines of "Army and Navy Notes" and is closed by the regularly published notice of "Orders Issued, etc." It was either written in all seriousness to portray the conception of this question from the view-point of the Medical Corps or it was left to the reporter to compose what he thought. Having been twice thus treated by these gentlemen, let us look over the situation and discern what motives prompt them to this action.

A few pertinent questions we might consider in this inspection are: How much latitude should the authorities at the head of the Medical Corps have? How really strong is this solicital supervision over the Dentists? Have these men the right to display their own selfishness in the exercise of an authority which does not rightfully belong to them? Who is logically in the position to restrict the Surgeon General? Who could dictate to the head of a medical organization about medical matters?

Modern progress is continually changing diagnosis, scientific methods are in many instances reversing old procedures, invention, discovery, research and economy are daily changing the best facilities for the Medical Corps. Who, then, has the right to dictate to the Surgeon General?

Would you involuntarily conceive that the heads of this great department, these magnanamous men, engrossed (as they are believed to be) in maintaining the efficiency of their Services for the prevention of disease, saving of lives and administering comfort in the Army and in the extremity of war, would you accredit to them an act of selfishness? Dental Surgeons in the Army now exist only under "Contract Services" which is the "Reserve." Naturally, under this condition, the nearest "Department Head" is the Surgeon General, which means that at times the Dental Surgeon is under the "command" of the lowest Medical Reserve officer.

All of this in retrospect seems very reasonable, but for the future, how can the necessarily biased view-point of the Medical Corps (both politically and practically) be altered to recognize even the immediate needs of a Dental Surgeon?

Any truthful physician will acknowledge that he knows about as little about dental practice as any layman. The high scientific plane on which the science of dentistry has placed its practice is only equaled by the additional scope of its possibilities.

The subservient position in our Army of the Dental Surgeons, being so completely under the dictation of the Medical Corps, that it literally carries a dead-weight and they labor so under various embarrassments.

So annoying are the ever-present importunities of the Medical Corps that it is always with great difficullty that requisitions for medicines and materials which are absolutely needed, are granted, as all these have to be secured over the signature of the nearest medical surgeon. So optional is the authority of these men, most of whom exercise their full dictatorial right, that criticisms, and substitutes are suggested for certain articles, the uses of which they do not know a thing about; or they are sometimes absolutely refused.

It is humiliating to know the truth, that the personal accounts of the Dental Surgeons show the purchase of absolutely necessary articles for practice which have been refused them by their wise guardians.

Under present conditions the Dental Surgeons are in ratio about one to every 5,000 soldiers, each territory including widely separated posts to which the Dental Surgeons have to carry their portable equipments. The percentage of soldiers under each Dental Surgeon being fully three times greater than the number which they can attend with any degree of satisfaction, combined with the numerous changes to which they are subject, greatly handicaps their best efforts for service.

An Army Medical Surgeon enters with the rank of 1st

Lieutenant, one promotion allowance ahead of the line officers, in consideration of the necessary college course for his professional education.

Each new enlistment (every three years) receives promotion consecutively up to their Majority. This I believe is correct or generally so. The preparation and knowledge that is required of them, their great importance cannot be discounted, but, their work is practically a fixed process, the carrying out of ordered methods, under the best preparation in facilities which can be supplied them.

These above facts are only cited for the purpose of comparison with those of the Dental Surgeons, and the field of their duties under a properly managed dispensation, which it is only fair that they should receive.

Capable dentists, especially such as are to meet out the social and other positions of Army life, must first possess the essential qualities of manhood, and be refined gentlemen.

The education prior to his dental course, to say the least must be good, and the diversity in the nature of the different subjects which he has to master, so as to blend them in the combination of his different duties, requires the most careful preparation.

The National Association of Dental Examiners requires of all institutions who issue recognized diplomas, a three year course of study and each year must comprise a 32 weeks session.

Anatomy, physiology, histology, pathology and bacteriology are absolutely essential studies to give him his ground work in the field of his operations just the same as the physician.

The dental course includes the essentials of general surgery and materia medica, as applied to the immediate needs of dental practice. At this the dental course has only reached the point at which it begins to specialize.

Chemistry, metallurgy, the applied sciences and art in the constructions to supply the losses of nature, operative dentistry, the mastery of all, and the ever increasing progress to be kept up, calls for men of resolution and scientific skill for our army purposes.

When a surgeon enters the army he receives his commission, quarters, retirement and a steady advance of promotion with no initiative work in his own hands.

A dentist, who has prepared as hard for entrance, receives only his contract, quarters, has hard initiative work in a much larger territory with its exacting activities, and there he remains, with only considerative advance in salary after certain age in service and privileges for outside practice after certain hours, which cannot be extensively reckoned of much value, having to travel so much. The Dental Surgeon is quartered in the line as a Lieutenant, there he remains to see medical men move up into Captains quarters, etc. What distinctive right has the medical man to be more favored?

This point, no doubt, is keenly felt in the personnel of army life and is worthy of mention. The dentist deserves the equal social right.

The Dental Surgeon, by every right in surgery, is the logical Oral Surgeon in the Army. He does not need the sarcastic "Automobile Dental Chair" of our appended maligners, but he can be a very busy man beside the field of battle, doing a service that no Medical Surgeon can perform, with his surgical equipment he can attend all fractured jaws and other wounds in the oral vicinity with the best of skill assuming the work which is necessarily slow and stubborn in healing and not always accompanied with good results.

The Dental Surgeon in the Army *must* be a man who can render any kind of service to the surgeons in the way of assistance when not employed in his own particular field.

The proper readjustment of the Medical and Dental relations has now become a paramount issue. It is not a matter of trivial importance, it is not an issue emanating from the minds of a few men or from a weak organization.

Delegates from the National Dental Association as our National Representatives are presenting to Congress the present conditions and petitioning for a proper re-organization. They have adopted plain measures of presenting the facts only which make clear the needs in proper form according to those who are in the best position to judge and have recommended specific bills to be discussed and voted upon in regular form.

Bills have been started through Congress with no special care or preparations to avoid the pit-falls of the designing ones whose favor they did not possess.

Twice they received proper attention from those to whom they were referred, i. e., the legislative bodies; but while passing through some of the dark corridors of "form" they became so misguided as to be finally lost, mislaid, by those prepared and make a political and not a meritorious account of where they were last seen.

Twice has the "sting" been applied and this is quite enough to locate the source. Let us take warning and benefit from our first two bills and prepare for a good and proper protection of the next.

The very nature of the constituents of dentistry with its eminent and representative men, and the nature of the national organization itself, makes the National Dental Association a stronger force than the Medical Department of the Civil Service.

The writer happens to be acquainted with a very interesting happening which occurred in one of our large cities last fall, the description of which will in no manner disclose the location or the parties involved. The Dental Society of that city sent a letter to the representative from its district, who was a candidate for re-election, explaining in a fair manner, the needs and the values and as well as the economical good that would accrue to our Army system, upon the proper re-organization of a Dental Corps.

They presented the evidence, that convinced them that their cause was right, and gave him an opportunity to examine it, and assured him if his views coincided with theirs in this matter, they would one and all pledge themselves to his support in the approaching election. This representative in Congress, was not on the stand for his first re-election, neither was he for his last, but he is one of the best and most highly respected among that great body. He replied, acknowledged the need and the merits of their proposal and assured his support in any official capacity that might come to him, promising his vote and influence in its behalf.

The remaining facts are that every one of these dentists' votes were needed in carrying the largest county of his district. Whether he would have received them otherwise is not known, but the facts are that he did receive them and they meant business to him.

In a later expression he was heard to state that the dentist represented that type of gentleman who had the ability to judge for themselves and were consequently, very hard to reach politically, but once had, they represented an advance of just that much over the ratio of votes obtainable by any other politically machined means of any opposition.

This form may be termed, well-earned, or deserved politics, this form is honest, reciprocating kind of politics, like all should be. The kind which defeated our two bills, is more of that "applied" or "inside" sort, gained or taken advantage of by some ascendency in other unlimited rights, closely associating the other, but if truly discovered would better be described as over-reaching, selfish politics.

Which of these is generally likely to win? The one best bet is that the "honest" method will be there at the best time, especially after having found the "inside" method's hiding place.

Next year the National Dental Association should use two of its committees to protect and prepare for the passage of its Bill. The Committee on Army Legislation will again be the active body for the drafting and presentation of the Bill and the committee and State, and Local Societies should arrange to take up a National correspondence with every Senator and Representative for the means of establishing their interest and support.

A member of either the National, State or Local Society should be selected in every Congressional district to first communicate with every dentist in his district, then present the proposal and petition of support to their Congressman by the best plan that may be selected by the National Association's Committee in charge of the work.

Since that part of this article was written referring to the Dentist in the Army, as being under contract services, certain changes have been made in the dispensation of the law, which commissions them with the rank of 1st Lieutenant.

There are a few advantages in this, namely: retirement at two-thirds pay, other than this, the possession of a sword and pistol represent the only other advantages gained.

The Committee of the National Dental Association on Army Dental Legislation made the most careful preparations last year for the proper passage of its Bill, but it was defeated by the base intrigues of the Medical Department. Their Bill asked for a Dental Corps with the progressive ranks up to and including Majors, with certain other very beneficial additions.

The Bill was appended as a "rider" on the Army Appropriation Bill and for three times it successfully passed the Senate.

Not at all disheartened but diligently and in every way possible, the National Association Committee is still working for the accomplishment of its objective; and it behooves us, one and all, to gladly respond to any requests made of us by the Committee, so that harmoniously, our cause, the great need of which being only too apparent, may in an upright and honest manner, over-ride the forces of all opposition and at an early date become a law.

### DISCUSSION.

Dr. A. J. Cottrell, of Knoxville: It would seem that in our desire to advance the interest of our profession that we have willfully neglected one of our greatest opportunities, simply because of the fact that the Army and Navy are somewhat removed from us, and that the services of the dentist is supposed to render in the Army and in the Navy are at posts of which we know nothing, many of them across the seas. As a profession we have not given this matter the thought that it deserves, and we have certainly failed to support our committee in this national work. At the meeting of the Southern Branch in Atlanta, a few days ago, Dr. Crenshaw presented this matter to the members of the Association and he called upon those members who were willing to actually do something when called upon by their committee, to rise up in their seats, and he took down their names. He said that he did not know when they would call upon them to do this work, but this is where you must show your interest, and nothing will be accomplished unless you do. A great number of men pledged themselves there to carry out whatever directions are given by that committee, and I tink it will be well enough for us to pledge to this committee our support in the work that they have undertaken. Now the one thing that impresses me most in the paper of Dr. Eby is the contempt and lack of regard that the medical profession has for us as a profession. I am confident it is because the great majority of medical men still remain ignorant of the services we render humanity; they still want to regard us as something inferior to themselves, and I think it will be well for this Association to take up this matter and give it some thought and consideration, and be prepared to intelligently assist in this work when asked. It seems to me that in this way we can do more to advance the general interests of our profession than in any other particular that we have at present.

Dr. Gordon White: I have not been a member of this committee for several years, I had the honor of being, however, on the original committee and we did a tremendous amount of work, directed very greatly by our enthusiast on the subject, Dr. Donnelly, who was then secretary of the committee appointed by the National Association at Old Point Comfort. I do not think I ever did in my experience in the profession such hard work. Much of the detail I knew nothing of, however, because it was managed by Dr. Donnelly and others at Washington City. It was through our influence that we got the first law through. Dr. Donnelly is not now connected with the committee, but he is still doing I think the best work he has ever done. What I have done for the profession, was done for the profession, and not for myself. and I am perfectly willing to continue working for the Association. The opposition of the medical department was not as great then as it is now. Just why I am not in a position to tell. There were a sufficient number of surgeons in the Army, however, who recognized the great need of dental surgery. They were, of course, anxious to have their soldiers better fitted for the exigencies of war, and there were also a number of people in Washington who were in favor of us. When we took up the subject of trying to get into the Navy, we found it a very different thing. We encountered considerable opposition there from all sources and of the medical department particularly, as Dr. Eby tells us, and the arguments opposed to us were in many instances ridiculous; it appeared that we knew nothing and that we were only carpenters.

The neglect that the profession at large has given these committees is incomprehensible. When I was serving actively on the committee, we spent much time writing to the prominent dentists all over the country, and it is simply astonishing to find how few of them entered into the fight or gave us any help or advice. I do not know to save my soul what we would have done if it had not been for the persistent work of Donnelly, who is an enthusiast on this subject. Scarcely a day passed that I did not write a letter to some senator or congressman, and to dentists all over the country, soliciting their aid, and you can't conceive of the little amount of interest we get from the profession at large. Some informed me, we were pushing our cause too fast and that we should wait until the authorities in the navy and army saw the need of it and they would call for us. I am still working at this matter even though I am not a member of the committee. I spoke to a senator only a few days ago in regard to it and I saw a physician and a congressman, and asked them to add their influence to our cause. I am hammering at it all the while. I do not know what the details are now, but I hear from Donnelly nearly every month and Donnelly is doing all he can to get the Bill through.

The jealousy of the medical men in the army is to my mind amazing. They do not want us to trespass upon their preserves. They do not want us there, we have never made a fight or approached the subject with the authorities that we haven't had a fight from that quarter. That was so in the old days, I know it is so now. Not only that, but there is jealousy in all branches of the army. Every officer seems to live in hopes of some superior dying in order that an order of promotion may go down the line. All of these things have always been disgusting to me. I think that we should be placed on an equality with the medical profession.

Dr. Crutcher: I want to thank Dr. Eby for his paper, and to say to him that I know something of his cause from reading about it. I suppose that jealousy in the army is like it is in some of our smaller towns between the physician and the dentist, we have all seen that more or less, and I suppose it exists to a cetrain extent in the cities. Some medical men, however, recognize the dentists for their work. The dentist has a large part in regard to health and relief of suffering humanity, almost as important as the physician. It is impossible for the physician to meet all the demands of ill health in all parts of the body and their should be a hearty co-operation of physician and dentist where the patient's welfare needs it. The troubles that exist in the army are all new to me. I would not suppose that in the army the physician would be hampered in any way when it comes to the treatment of any ills of a member of the army, and I feel that the dental practitioner in the army should have the same opportunity and help as the physician. I feel that our Association ought to go on record as cordially approving this bill and as individuals let us do whatever we can to assist the Committee in furthering this cause in the army. I think we ought, like Dr. White, to appeal to our congressmen and senators. Every man has an influence, and if we can get the six hundred dentists in Tennessee to urge our eleven or twelve congressmen and senators to act for us we can do a good work. If other states interest their congressmen the time will come when our men will be recognized in the army and the navy as much as the medical men.

A Member: The trend of the discussion is that each individual do something, but I understand from Dr. Crenshaw that we should do nothing until the committee calls upon us to do what the committee desires, as they can put their fingers upon every man favorable to the Bill and when the committee gets ready they will call upon us to work with certain individuals.

Dr. Eby: The plan of work agreed upon is to try to conduct everything as harmoniously and uniformly as possible and in a systematic way, so that no discord will arise and no field of argument would be brought forward by circumstances and information which may be gathered by those with whom we talk. They plan to correspond with every man in every congressional district where work is needed and they want you to wait and be ready for orders.

